# Factors Associated With Composite Anthropometric Failures (CIAF) Among Under Five Children in Lesotho: An Insight From the 2023 to 2024 Demographic and Health Survey Data

**DOI:** 10.1002/fsn3.71607

**Published:** 2026-03-04

**Authors:** Nigussie Adam Birhan, Denekew Bitew Belay

**Affiliations:** ^1^ Department of Statistics, College of Natural and Computational Science Injibara University Injibara Ethiopia; ^2^ Department of Statistics, College of Science Bahir Dar University Bahir Dar Ethiopia; ^3^ School of Health Systems and Public Health, Faculty of Health Sciences University of Pretoria Pretoria South Africa

**Keywords:** composite index for anthropometric failure, Lesotho, under five children

## Abstract

Under nutrition is the main cause of child death in developing countries. The Composite Index of Anthropometric Failure (CIAF) combines all three forms of anthropometric failures to assess the nutrition status of children. Thus, the objective of this was to identify factors associated with CIAF of under‐five children in Lesotho. A secondary analysis of the Lesotho Demographic and Health Survey 2023–24 was conducted, using the data for 1089 children under the age of 5 years. The CIAF was used to classify children based on stunting, wasting, and underweight. Descriptive summary statistics were computed. A binary logistic regression model was employed to identify predictors of CIAF for under‐five children. Adjusted odds ratio with 95% confidence interval was estimated. The prevalence of CIAF in Lesotho was 34.68% (95% CI: 31.76–37.73). Female child 0.54 (AOR = 0.54; 95% CI: 0.375, 0.776), age group 24–59 months 2.42 (AOR = 2.42; 95% CI: 1.149, 5.109), rich households 0.29 (AOR = 0.29; 95% CI: 0.151, 0.554), multiple births 12.02 (AOR = 12.02; 95% CI: 1.199, 120.426), rural residence (AOR = 0.56: 95% CI: 0.335, 0.946), living children 3 to 4 were 2.54 (AOR = 2.54; 95% CI: 1.522, 4.226), and larger size at birth were 0.38 (AOR = 0.38; 95% CI: 0.211, 0.683) were found to be significantly associated with CIAF. The prevalence of CIAF among children under five in Lesotho was high. Child's age, child's sex, child's type of birth, wealth tercile, residence, number of living children, and child's birth size were found to be significantly associated with CIAF. We suggest that the government adapt CIAF as a metric for assessing children's nutritional status in order to estimate the overall prevalence of malnutrition and strengthening adequate nutrition intervention programs in rural areas. Furthermore, highlighting the factors influencing child CIAF will help inform future policies and programs designed to approach this major problem in Lesotho.

## Graphical abstract

Prevalence of seven categories of CIAF classification of nutritional status among children under five in Lesotho. This study assessed anthropometric failures among under‐five children in Lesotho using the Composite Index of Anthropometric Failure (CIAF). The prevalence of the Composite Index of Anthropometric Failure (CIAF), stunting, wasting, and underweight was 34.68%, 23.54%, 0.39%, and 0.63%, respectively and significant predictors included child's age, sex, type of birth, wealth status, residence, number of living children, and birth size. These findings highlight the importance of adopting CIAF as a comprehensive indicator of malnutrition and inform policy for targeted nutrition interventions in Lesotho.

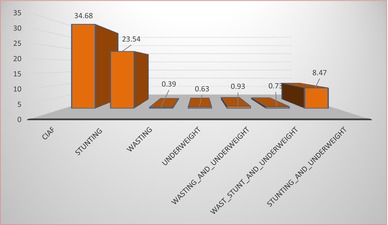

**Abbreviations**
CIAF: composite index of anthropometric failure
DHS: Health and Demography Survey
LHDS: Lesotho Health and Demography Survey
WHO: World Health Organization

## 1 Introduction

Child malnutrition is the major public health problem in the world, given that an estimated 45% of all deaths among under five children are attributed to child malnutrition (Talukder 2017). Child undernutrition is the most common form of malnutrition and a major contributor to child mortality in low–middle income countries (Rasheed and Jeyakumar 2018; Asmare et al. 2025). Beyond child survival, child malnutrition has implications for cognitive and psychosocial development of the child, and the economic productivity of individuals and societies (Candler et al. 2017; Birhan and Belay 2021; Tegegne and Belay 2021; Alamirew et al. 2022; Debelu et al. 2021).

Child under nutrition is a major public health issue in developing countries, ranked as a top global challenge with severe human and economic impacts, especially on the poor, women, and children (Muchie et al. 2022; Mannar et al. 2020). In 2020, an estimated 149 million (22%) children under five worldwide were stunted, and 45 million (7%) were wasted (WHO 2022). In sub‐Saharan Africa, malnutrition has increased from 5.5 million to 30 million in the past decade, contributing to more than 3.5 million deaths of children under five annually (Owolade et al. 2022; Agidew et al. 2023). The region bears the highest burden of under nutrition compared to other sub‐regions due to poverty, food insecurity, political instability, climate variability, inadequate infrastructure, and poor feeding practices (Roche 2023; Enbeyle et al. 2022; Warssamo et al. 2025).

Lesotho has designed and implemented the linking Food Security to Social Protection Programme (LFSSP) which is a government‐led, multi‐sector initiative integrating health and agriculture efforts to combat under nutrition. It promotes child feeding, dietary diversity, and nutrition behavior change through interventions such as homestead gardening training, seed distribution, and nutrition education for vulnerable rural households (Dewbre et al. 2015). However, under nutrition remains a significant public health issue in Lesotho, with a persistently high burden of both acute and chronic malnutrition. According to the Lesotho Demographic and Health Survey (LDHS) of 2009, 2014, and 2023–24 the prevalence of under‐five stunting was 39%, 33%, and slightly increasing 36% respectively (DHS 2024).

Previous studies on under nutrition prevalence and its associated factors have typically focused on a specific indicator, such as stunting, wasting, and underweight using conventional nutritional assessment methods (Rasheed and Jeyakumar 2018; De Onis et al. 2012; Nandy and Svedberg 2012; Leseba et al. 2025). However, children who are underweight may experience stunting and/or wasting, and some children may suffer all three anthropometric failures simultaneously. Therefore, none of these conventional nutritional indicators are able to precisely characterize the overall burden of under nutrition among children under the age of five. To address this issue, the composite index of anthropometric failure (CIAF), a multidimensional single index, was introduced by Svedburg and Nandy in 2000 which may show signs of having two or more anthropometric failures simultaneously (Svedberg 2000; Nandy et al. 2005).

The CIAF offers a comprehensive view of childhood under nutrition in resource‐limited settings by combining various categories of anthropometric failures such as wasting only; wasting and underweight; wasting, stunting, and underweight; stunting and underweight; stunting only; and underweight only. It is widely recommended as a reliable measure of malnutrition, helping to identify multiple nutritional deficiencies through a single, aggregated indicator (Nandy et al. 2005; Balogun et al. 2021; Islam and Biswas 2020). Literature supports the use of CIAF rather than using traditional methods for the assessment of children under nutrition status (Preedy 2012). This study provides new estimates for the prevalence of under nutrition by aggregating traditional under nutrition indices, which are important to capture the overall impact of under nutrition on a population unlike that of any of the three traditional indicators. To address the above identified gaps, this study aims to assess under nutrition among children under the age of five in Lesotho using the CIAF and to identify the factors associated with it.

## 2 Methods and Materials

### 2.1 Study Design and Settings

A cross‐sectional study design was employed. The sampling frame used for the 2023–24 LDHS is based on the 2016 Lesotho Population and Housing Census (2016 PHC), conducted by the Lesotho Bureau of Statistics. Lesotho is administratively divided into 10 districts; each district is subdivided into constituencies and each constituency into community councils.

### 2.2 Data Sources and Study Population

The 2023–24 Lesotho Demographic and Health Survey (2023–24 LDHS) is the fourth nationally representative DHS survey conducted in Lesotho. Data collection took place from 27 November 2023 to 29 February 2024. The data set used in this study is freely available and possible to download by justifying the reason for requesting the data from the link: https://dhsprogram.com/data/available‐datasets.cfm.

#### 2.2.1 Sample Size and Sampling Procedure

**FIGURE 1:**
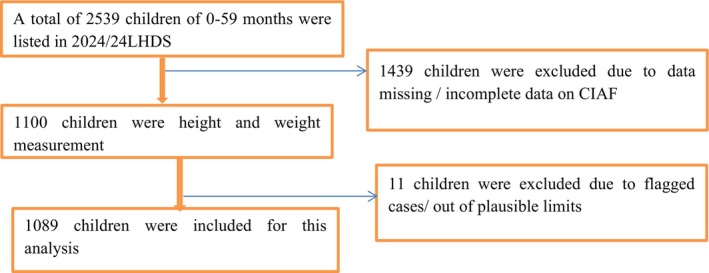
Schematic diagram for the sample selection in Lesotho.

The DHS is a community‐based, cross‐sectional survey conducted every 5 years, aimed at producing health and health‐related indicators for low‐ and middle‐income countries. It employs a two‐stage stratified cluster sampling design, where enumeration areas serve as the first stage, and a sample of households is drawn from each enumeration area in the second stage. In the 2023–2024 LDHS, mothers/caregivers were the primary respondents for questions related to child health and nutrition. In the second stage of selection, 25 households per cluster (EA) were systematically selected with equal probability selection from the newly created household listing, *all* eligible women aged 15–49 in the selected households were interviewed. Anthropometric data (weight, height/length) were collected directly from all children under five living in those households, but the questionnaire responses were provided by their mothers or primary caregivers. A total weighted 1089 under five children were included in the analysis that accounted for clustering by using survey weights (Figure 1).

#### 2.2.2 Study Variable

In this study the outcome variable was a CIAF among children coded as 1 = failures, 0 = not a failure. Children who did not show any signs of anthropometric failure from categories B to Y were classified as “no failure”. The purpose of CIAF is to identify all the undernourished children and to measure the total burden of child malnutrition on the population. CIAF helps disaggregate malnourished children into different subgroups. B, F, and Y are the groups of single‐burden anthropometric failure. E and C are the groups of double‐burden of anthropometric failure. Group D is the group of triple‐burden of anthropometric failure.

According to CIAF standards, a child was classified as having “anthropometric failure” if the child exhibited any of the deficiencies listed in categories B to Y as it is clearly depicted in (Table 1).

**TABLE 1:** Category of anthropometric failure in under five children using Composite Index of Anthropometric (CIAF).

Groups	CIAF categories	Description of the level	Wasting	Stunting	Underweight
A	No failure	Normal WAZ, HAZ and WHZ	No	No	No
B	Wasting only	WAZ < −2SD, but normal HAZ and WHZ	Yes	No	No
C	Wasting, underweight	WAZ and WHZ < −2SD, but HAZ normal	Yes	No	Yes
D	Wasting, stunting and underweight	WAZ, WHZ and HAZ < −2SD	Yes	Yes	Yes
E	Stunting, underweight	HAZ and WHZ < −2SD, but WAZ normal	No	Yes	Yes
F	Stunting only	HAZ < −2SD, but normal WAZ and WHZ	No	Yes	No
Y	Underweight only	WHZ < −2SD, but normal HAZ and WAZ	No	No	Yes

This study examined several independent variables influencing composite index of anthropometric failure selected by reviewing related work of the literature (Ayres et al. 2024; Fenta et al. 2021). Maternal education, employment, place of residence, current marital status, age at first birth, preceding birth interval, number of living children, and media exposure were included as independent variables related to the mother. Independent variables related to the child included in the analysis were: age, sex, place of delivery, type of birth, birth size, breast feeding status, and experience of diarrhea in the last 2 weeks, vitamin A supplementation. The household variables were: wealth tercile, source of drinking water, and toilet facility.

### 2.3 Data Management and Analysis

The data were cleaned using STATA 18. The survey set function in Stata was used to identify the strata, the primary sampling units, and the sampling weight so that the stratified, clustered study design was taken into account when calculating standard errors to get reliable statistical estimates. Descriptive and summary statistics were conducted. A binary logistic regression model was used to assess the associations between dependent and independent variables. Variables with *p*‐value < 0.25 in bivariable analysis were included in the multivariable logistic regression analysis to compensate for confounders. Adjusted odds ratios with 95% confidence interval were reported, and significance at *p*‐value < 0.05.

### 2.4 Ethics Statement

We used datasets provided by the Demographic Health Surveys program and have not had any form of contact with the study participants. Informed consent for the present analysis was not necessary because secondary data analysis did not involve interaction with the participants. Ethical clearance for the Demographic Health Survey (DHS) was provided by the Lesotho Health and Nutrition Research Institute (LHNRI) Review Board, the National Research Ethics Review Committee (NRERC) at the Ministry of Science and Technology, the Institutional Review Board of ICF International, and the CDC. Further information regarding the DHS data usage and ethical standards can be accessed online at https://dhsprogramcom/data/AccessInstructionscfm.

## 3 Results

### 3.1 Characteristics of the Study Participants

A total of 1089 weighted samples of under five children were included in this study. From this study finding 550 (50.51%) children were males. Of the total mothers, 707 (70.63%) of them were married. 451 (41.37%) of the total respondents were in the poor wealth tercile, among these 206 (45.79%) experienced CAIF. In terms of sanitation, 239 (21.93%) of respondents used non‐improved sanitation (Table 2).

**TABLE 2:** Background characteristics of the study participants.

Variables	Categories	Nourished count (%)	CAIF count (%)	Total count	Chi‐square statistic (*p*)
Age of child in months	0–5	108 (15.23)	34 (9.13)	143 (13.11)	5.28 (0.071)
	6–23	232 (32.64)	137 (36.29)	369 (33.91)	
	24–59	371 (52.13)	206 (54.57)	577 (52.98)	
Sex of child	Male	333 (46.88)	217 (57.35)	550 (50.51)	9.12 (0.002)
	Female	378 (53.12)	161 (42.65)	539 (49.49)	
Type of birth	Single	703 (98.84)	366 (96.99)	1069 (98.20)	14.37 (< 0.001)
	Multiple	8 (1.16)	12 (3.01)	20 (1.80)	
Breast feeding status	Ever breastfed	190 (26.74)	144 (38.06)	334 (30.67)	17.40 (< 0.001)
	Never breastfed	18 (2.59)	13 (3.53)	32 (2.92)	
	Still breastfeeding	503 (70.67)	221 (58.40)	723 (66.42)	
Place of delivery	Home	23 (3.24)	33 (8.84)	56 (5.18)	14.71 (< 0.001)
	Health facility	688 (96.76)	334 (91.16)	1033 (94.82)	
Diarrhea	No	566 (79.59)	313 (82.77)	879 (80.69)	**1.16 (0.281)**
	Yes	145 (20.41)	65 (17.23)	210 (19.31)	
Wealth tercile	Poor	244 (34.34)	206 (54.63)	451 (41.37)	41.83 (< 0.001)
	Middle	145 (20.38)	76 (20.22)	221 (20.33)	
	Rich	322 (45.28)	95 (25.16)	417 (38.30)	
Mother education	No education	2 (0.32)	2 (0.52)	4 (0.39)	26.24 (< 0.001)
	Primary	162 (22.75)	126 (33.41)	288 (26.45)	
	Secondary	448 (65.58)	235 (62.24)	683 (62.72)	
	Higher	99 (13.97)	14 (3.84)	114 (10.45)	
Age at first birth	Less than 18	117 (16.51)	94 (24.97)	212 (19.45)	16.64 (< 0.001)
	18 and above	594 (83.49)	283 (75.03)	877 (80.55)	
Mother employment status	Not working	474 (66.66)	279 (73.87)	753 (69.16)	3.72 (0.054)
	Working	237 (33.34)	99 (26.13)	336 (30.84)	
Drink water sources	Not improved	114 (16.07)	72 (18.95)	186 (17.07)	**0.96 (0.327)**
	Improved	597 (83.93)	306 (81.05)	903 (82.93)	
Toilet facility	Not improved	139 (19.50)	100 (26.50)	239 (21.93)	9.09 (0.003)
	Improved	572 (80.50)	278 (73.50)	850 (78.07)	
Media exposure	No	163 (22.96)	134 (35.46)	297 (27.30)	12.33 (< 0.001)
	Yes	548 (77.04)	244 (64.54)	792 (72.70)	
Residence	Urban	276 (70.77)	114 (29.23)	390	11.21 (0.001)
	Rural	435 (62.27)	264 (37.73)	699	
Vitamin A supplementation	No	285 (40.06)	145 (38.44)	430 (39.50)	1.94 (0.164)
	Yes	426 (59.94)	233 (61.56)	659 (60.50)	
Health care facility	Not big problem	493 (69.25)	203 (53.82)	696 (63.89)	8.16 (0.004)
	Big problem	219 (30.75)	174 (46.18)	393 (36.11)	
Living children	1–2	556 (78.20)	230 (60.79)	786 (72.16)	18.18 (< 0.001)
	3–4	128 (17.93)	111 (29.48)	239 (21.94)	
	5 and above	28 (3.87)	37 (9.73)	64 (5.90)	
Birth interval	First	321 (45.13)	131 (34.58)	452 (41.47)	14.61 (0.002)
	Less than 33 month	69 (9.70)	62 (16.32)	131 (11.99)	
	33–59	133 (18.75)	85 (22.63)	219 (20.09)	
	Above 59 month	188 (26.42)	100 (26.47)	288 (26.44)	
Marital status	Unmarried	118 (16.63)	53 (14.09)	171 (15.75)	**1.93 (0.380)**
	Married	503 (70.65)	267 (70.60)	770 (70.63)	
	separated	90 (12.72)	58 (15.31)	148 (13.62)	
Birth size	Smaller than average	53 (7.39)	49 (12.86)	101 (9.29)	9.53 (0.009)
	Average	531 (74.66)	279 (73.98)	810 (74.42)	
	Larger than average	128 (17.95)	50 (13.16)	177 (16.29)	

*Note:* The bold values indicate variables are not statistical significance with *p* < 0.25 in the bivariable analysis for inclusion in the multivariable model.

### 3.2 Prevalence of Childhood Under Nutrition

Table 3 shows the prevalence of under nutrition among the children under 5 years old in Lesotho. The prevalence of stunting, wasting, and underweight was 23.54%, 0.39%, and 0.63%, respectively. The overall prevalence of under nutrition using the CIAF among the children under 5 years old was 34.68% (31.76, 37.73).

**TABLE 3:** Distribution of children under 5 years according to the seven categories of CIAF classification of nutritional status, based on data obtained from surveys 2023 to 2024 conducted in Lesotho.

CIAF categories		Count	% (95% CI)
A	No failure	711	65.32 (62.27, 68.24)
B	Wasting only	4	0.39 (0.14, 1.05)
C	Wasting, underweight	10	0.93 (0.49, 1.78)
D	Wasting, stunting and underweight	8	0.73 (0.35, 1.51)
E	Stunting, underweight	92	8.47 (6.88, 10.38)
F	Stunting only	256	23.54 (20.99, 26.31)
Y	Underweight only	7	0.63 (0.29, 1.38)
	CIAF (%)	378	34.68 (31.76, 37.73)

### 3.3 Multicollinearity Test

All predictors had VIF values ranging from 1.12 to 2.18 (mean VIF = 1.46), indicating no evidence of multicollinearity (Table 4).

**TABLE 4:** Multicollinearity assessment of predictor variables using VIF.

Variable	VIF	1/VIF
Wealth Index	2.18	0.459
Toilet facility	1.49	0.671
Residence	1.74	0.574
Mother education	1.76	0.567
Age of child in months	1.65	0.605
Media exposure	1.50	0.666
Living children	1.45	0.690
Birth interval	1.43	0.698
Health care facility	1.41	0.712
Breast feeding status	1.40	0.712
Type of birth	1.38	0.723
Place of delivery	1.30	0.767
Vitamin A supplementation	1.30	0.769
Mother employment status	1.30	0.771
Age at first birth	1.20	0.830
Child at birth size	1.16	0.859
Sex	1.12	0.889
Mean VIF	1.46	

### 3.4 Factors Associated With the CIAF

In univariable analysis, diarrhea, source of drinking water, and marital status were not significantly associated with CIAF at a *p*‐value < 0.25 and excluded from the multivariable logistic regression analysis (Table 2). The CIAF was significantly associated with the child's age, child's sex, child's birth type, wealth tercile, residence, living children, and child's birth size in a multivariable logistic regression model (Table 5). The odds of having CIAF were lower among females 0.54 (AOR = 0.54; 95% CI: 0.375, 0.776) as compared to males keeping the other covariates constant. The odds of having CIAF were higher among under five older age groups 24–59 months 2.42 (AOR = 2.42; 95% CI:1.149, 5.109) as compared with those whose ages were below 6 months. Under five children from rich households had 0.29 (AOR = 0.29; 95% CI: 0.151, 0.554) times lower odds of CIAF as compared to those who were from poor households. The odds of having CIAF among multiple births were 12.02 (AOR = 12.02; 95% CI: 1.199, 120.426) times higher than those of single births.

**TABLE 5:** Factors associated with CIAF in children under 5 years old.

Variables	Categories	AOR (95% CI)	*p*
Age of child in months	0–5 (ref)	1	
	6–23	1.69 (0.904, 3.151)	0.100
	24–59	2.42 (1.149, 5.109)	0.020*
Sex of child	Male (ref)	1	
	Female	0.54 (0.375, 0.776)	0.001*
Type of birth	Single (ref)	1	
	Multiple	12.02 (1.199, 120.426)	0.034*
Breast feeding status	Ever breastfed (ref)	1	
	Never breastfed	1.02 (0.428, 2.410)	0.971
	Still breastfeeding	0.67 (0.412, 1.080)	0.099
Place of delivery	Home (ref)	1	
	Health facility	0.58 (0.294, 1.138)	0.113
Wealth tercile	Poor (ref)	1	
	Middle	0.61 (0.367, 1.019)	0.059
	Rich	0.29 (0.151, 0.554)	< 0.001*
Mother education	No education (ref)	1	
	Primary	0.06 (0.001, 6.121)	0.229
	Secondary	0.88 (0.558, 1.376)	0.565
	Higher	0.62 (0.281, 1.372)	0.239
Age at first birth	Less than 18 (ref)	1	
	18 and above	0.89 (0.571, 1.376)	0.592
Mother employment status	Not working (ref)	1	
	Working	1.05 (0.667, 1.655)	0.831
Toilet facility	Not improved (ref)	1	
	Improved	0.83 (0.520, 1.331)	0.443
Media exposure	No (ref)	1	
	Yes	0.83 (0.537, 1.270)	0.383
Residence	Rural (ref)	1	
	Urban	0.56 (0.335, 0.946)	0.030*
Vitamin A supplementation	No (ref)	1	
	Yes	0.80 (0.517, 1.231)	0.307
Health care facility	Not big problem (ref)	1	
	Big problem	1.24 (0.848, 1.820)	0.265
Living children	1–2 (ref)	1	
	3–4	2.54 (1.522, 4.226)	< 0.001*
	5 and above	1.93 (0.852, 4.389)	0.115
Birth interval	Less than 33 month (ref)	1	
	First	1.06 (0.570, 1.658)	0.863
	33–59	1.04 (0.552, 1.971)	0.897
	Above 59 month	0.83 (0.448, 1.537)	0.552
Birth size	Smaller than average (ref)	1	
	Average	0.61 (0.370, 1.000)	0.050
	Larger than average	0.38 (0.211, 0.683)	0.001*

* Indicates statistical significance at the *p* < 0.05.

The odds of children having a CIAF were decreased by about 44% for children from urban areas compared to that of rural residents (AOR = 0.56: 95% CI: 0.335, 0.946). The odds of having CIAF for a household of living children 3 to 4 were 2.54 (AOR = 2.54; 95% CI: 1.522, 4.226) times higher compared to 1 to 2 living children. The odds of CIAF among larger sizes at birth were 0.38 (AOR = 0.38; 95% CI: 0.211, 0.683) times higher compared to small sizes at birth.

## 4 Discussion

This study revealed that 34.68% [95% CI: 31.76–37.73] of children had CIAF in the 2023–2024 national survey. The result of this study is in line with studies conducted in West Bengal of India 32.7% (Dasgupta et al. 2014) and higher compared to a study in Argentinians (15.1%) (Bejarano et al. 2019). However, this is lower than studies conducted in Yemen 70.1% (Al‐Sadeeq et al. 2018), Bangladesh 48.3% (Islam and Biswas 2020), and southern India 58.59% (Dhok and Thakre 2016). The variation in prevalence across these regions can be attributed to differences in socioeconomic conditions, healthcare access, and public health interventions, which influence child growth outcomes and rates of malnutrition.

This study indicated that children in the older age groups had a higher risk of anthropometric failure than children in the younger age group. This finding was consistent with studies conducted in Tanzania (Khamis et al. 2020) and Yemen (Al‐Sadeeq et al. 2018). The failure may result from the termination of breastfeeding, an increment of nutritional demand during rapid physical and mental growth, a lack of adequate and balanced food intake to meet the metabolic demand for childhood growth as they age, and older children's frequent interactions with their unhygienic surroundings, which may increase the risks of exposure to childhood infectious diseases that increase the risk of childhood under nutrition (Seboka et al. 2021).

The odds of children experiencing a CIAF were lower for children from rich households than poor households. This finding is consistent with other studies (Das and Gulshan 2017; Anik et al. 2021). This could be because the wealthiest households can afford to buy different types and amounts of food for their kids and improving mothers' ability to afford the cost of healthy food and ensuring household food security, while poorer homes may have less access to health care services than wealthier ones (Ayres et al. 2024).

The result revealed that children living in rural areas are more likely to have CIAF compared to urban children. This is consistent with previous studies conducted in Myanmar (Kang and Kim 2019), Bangladesh (Endris et al. 2017). This could be because children in cities have better living conditions and easier access to food.

In this study, multiple births were more likely to have CIAF than their counterparts, and this is supported by a study conducted by Ethiopia (Belay et al. 2023). This may be due to children from multiple births, where inadequate breastfeeding and competition for nutrition are more common (Adekanmbi et al. 2013).

The findings showed that female children had a lower risk of having CIAF than male children. This study is consistent with previous studies (Fenta et al. 2021; Kassie and Workie 2020; Tesfaw and Woya 2022). It could be because male infants are biologically more vulnerable to growth and illness in early life and have a higher rate of preterm births compared to females (Dewey and Brown 2003; Svefors et al. 2016). Additionally, there is a perception that girls are less likely affected by environmental stress than boys (Aheto et al. 2015).

According to the findings, children with a higher number of living siblings were more likely to be CIAF. This study is consistent with a previous study (Fotso 2007). This may be resource dilution within larger households, where limited food, care, and attention are shared among more children. Furthermore, large birth size was less likely to have CIAF than small birth size, and this is supported by a study conducted in Bangladesh (Hassan et al. 2025) and Ethiopia (Fenta et al. 2021). It could be that smaller birth size often reflects poor maternal nutrition and inadequate prenatal care, both of which can hinder fetal growth and contribute to low birth weight (Marshall et al. 2022).

## 5 Strength and Limitation of Study

This study's strength is using a nationwide population‐based dataset that provides a large sample size and statistical power to identify the factors associated with CIAF. Due to the cross‐sectional nature of this study, the direction of associations cannot be firmly established. Moreover, this study employed secondary data; it did not account for factors that could affect the occurrence of CIAF, such as food security and health‐related factors. Finally, the recall bias might have occurred because of the self‐reported nature of some of the variables. Although this study employed binary logistic regression for clarity and interpretability, the hierarchical nature of DHS data suggests that future research should apply multilevel and spatial modeling approaches to better capture clustering and geographic variation.

## 6 Conclusion

The study highlights a high prevalence of under nutrition in Lesotho as measured by the CIAF, which comprehensively captures all forms of anthropometric failures. In this study, child's age, child's sex, child's type of birth, wealth tercile, residence, number of living children, and child's birth size were significantly associated with CIAF. This study emphasizes the urgent adoption of the CIAF within Lesotho's LFNP framework to improve malnutrition assessment and guide integrated interventions, especially in rural areas. The findings have been shared with key stakeholders, including the Ministry of Health and the Lesotho National Nutrition Committee, who are considering incorporating CIAF into national monitoring systems to inform policy, allocate resources, and design targeted nutrition programs.

We also suggest that the government adapt CIAF as a metric for assessing children's nutritional status in order to estimate the overall prevalence of malnutrition. Given the high scale of malnutrition in Lesotho, there is an urgent need to accelerate efforts to address the multiple anthropometric failures through a holistic approach such as double‐duty actions, which is an efficient way to tackle these coexisting forms of malnutrition simultaneously through nutrition‐specific and nutrition‐sensitive interventions. Furthermore, highlighting the factors influencing child CIAF will help inform future policies and programs designed to approach this major problem in Lesotho.

## Author Contributions

**Denekew Bitew Belay:** conceptualization, writing – review and editing, visualization. **Nigussie Adam Birhan:** conceptualization, investigation, methodology, validation, software, formal analysis, data curation, writing – original draft.

## Funding

The authors have nothing to report.

## Ethics Statement

The current study was built on the analysis of openly accessible secondary data with all identifier information removed. The IRB of ICF Macro complied with the United States Department of Health and Human Services requirements for the “Protection of Human Subjects” (45 CFR 46). sThe IRB approved procedures for DHS public use datasets do not in any way allow respondents, households, or sample communities to be identified. There are no names of individuals or household addresses in the data files. In addition, written informed consent was obtained from a parent or guardian for participants under 16 years old. DHS Program has remained consistent with confidentiality and informed consent over the years. We obtained express approval to use the data from ICF Macro. No further approval was required for this study. The data can be found at https://www.dhsprogram.com/data/dataset_admin/login_main.cfmm. Further documentations on ethical issues relating to the surveys are available at http://dhsprogram.comm. We confirmed that all methods were carried out in accordance with the relevant guidelines and regulations.

## Consent

The authors have nothing to report.

## Conflicts of Interest

The authors declare no conflicts of interest.

## Data Availability

The data underlying the results presented in the study are publicly accessible and available from the DHS website (https://dhsprogram.com/data/available‐datasets.cfm). The name of the dataset is Lesotho Demographic and Health Survey (LDHS) 2023–2024.
